# Graphene and Related Materials: Properties and Applications in Dentistry

**DOI:** 10.3390/ma18235365

**Published:** 2025-11-28

**Authors:** Teissir Ben Ammar, Tatiana Roman, Housseinou Ba, Vincent Ball, Naji Kharouf

**Affiliations:** 1 INSERM UMR_S 1121, CNRS EMR 7003, Université de Strasbourg, Biomaterials and Bioengineering, Centre de Recherche en Biomédecine de Strasbourg, 1 rue Eugène Boeckel, 67000 Strasbourg, France; 2 Blackleaf, 210 rue Geiler de Kaysersberg, 67400 Illkirch-Graffenstaden, France; 3 Faculté de Chirurgie Dentaire, Université de Strasbourg, 8 rue Sainte Elisabeth, 67000 Strasbourg, France

**Keywords:** graphene and related materials, biomolecule-mediated exfoliation, dental materials, dental applications

## Abstract

This review summarizes recent advances in biomolecule-mediated exfoliation of graphene and related materials, and discusses their emerging applications in dental medicine. Natural biomolecules, including polyphenols, proteins and polysaccharides, are evaluated as exfoliating agents, emphasizing their influence on the structural and biological properties of graphene and related materials. Particular attention is given to how the synthesis methodologies affects physicochemical properties of the resulting materials and, in turn, biological and mechanical properties. The practical relevance of these materials in dentistry is demonstrated through their applications as functional fillers in dental cements, luting agents, endodontic sealers, and restorative composites, as well as advanced protective coatings for dental substrates and devices. Mechanistic insights into how exfoliation-driven structural modifications dictate material performance in specific dental applications are provided. Collectively, the findings highlight that biomolecule-mediated approaches represent a sustainable, scalable, and versatile strategy for engineering graphene-based materials that simultaneously meet functional requirements and biocompatibility standards essential for successful dental applications.

## 1. Introduction

In 2010, Andre Geim and Konstantin Novoselov from the University of Manchester were awarded the Nobel Prize in Physics for their pioneering work on graphene, a two-dimensional material consisting of a single layer of carbon atoms arranged in a hexagonal lattice [1]. This recognition catalyzed research interest in graphene, driven by its extraordinary combination of mechanical strength (Young’s modulus~1 TPa), exceptional electrical conductivity (electron mobility > 15,000 cm^2^/V·s), superior thermal conductivity (~5000 W/m·K), and remarkable chemical stability [1,2,3,4]. These exceptional properties position graphene as a transformative material across diverse scientific and technological domains, with particularly promising applications in biomedicine [1,5,6,7].

In dentistry, these properties are important for developing advanced materials such as restorative composites with enhanced mechanical properties [8], bioactive dental cements with improved bonding characteristics [8], and protective coatings that provide antimicrobial and anticorrosive properties [8,9], all of which contribute to improved durability, infection control, and long-term treatment success [10,11,12,13].

However, translating these properties from the nanoscale to real integrated applications remains challenging. First, this is due to the wide variety of available graphene materials and their differing characteristics [14]. Second, this consequently introduces uncertainty as to whether the type of graphene tested in dental research is the most appropriate.

Indeed, many synthesis techniques are used to produce graphene, and the resulting material can present various advantages and limitations depending on the intended use [10,14,15]. In 2024, the International Organization for Standardization published the Technical Specification ISO/TS 80004-13:2024, which provides updated nomenclature and standardized terminology for graphene and related materials (GRM) [16]. This specification covers the broader GRM family) [11], spanning from single-layer graphene (1LG) to few-layer graphene (FLG, 3–10 layers), multilayer graphene (MLG, up to 10 layers), and graphene nanoplatelets (GNPs, up to 250 layers). It also includes derivatives produced through controlled oxidation/reduction processes [4,11] such as Graphene Oxide (GO) and reduced graphene oxide (rGO) [4,11].

**Figure 1:**
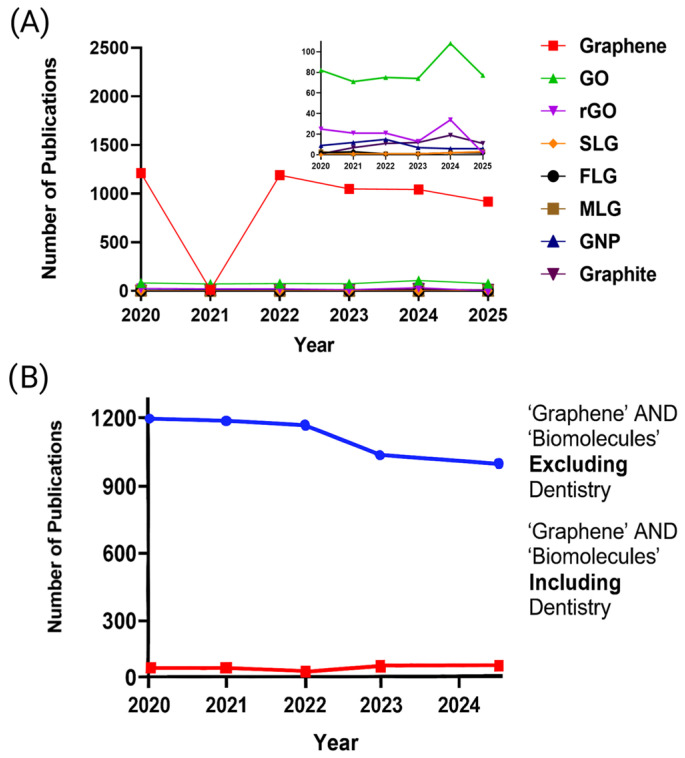
Bibliometric analysis (2020–2025) based on PubMed and Web of Science searches: (**A**) Distribution of publications by GRM type, with a magnified inset; (**B**) Publication trends based on the search terms ‘Graphene AND biomolecule,’ excluding and including dental research. The complete search strategy is detailed in Appendix A.

A bibliometric search conducted over the past five years (2020–2025) showed that the term ‘graphene’ remains the dominant descriptor in dental research (Figure 1), with graphene oxide (GO) clearly prevailing over other forms of GRMs in dental studies (The search strategy applied to the PubMed and Web of Science databases is detailed in the Appendix A.1). These findings support the observation that current dental literature has yet to fully adopt the updated GRM nomenclature or reflect the growing diversity of GRM now available on the market.

The prominence of GO in dental research is partly historical: it was one of the first graphene derivatives produced at scale through the Hummers method [17], which greatly accelerated its adoption, particularly following the 2010 Nobel Prize announcement. Its popularity in biomedicine also stems from its abundance of oxygen-containing functional groups (hydroxyl, carboxyl, and epoxy), which enhance hydrophilicity, and enable chemical modification [6,18,19]. However, most studies in dental research and biomedicine tend to overlook the environmental and scalability challenges associated with GO synthesis routes, issues that are well documented in fields such as energy storage, flexible electronics, and composite manufacturing [6,14,17].

In particular, the Hummers method is known to introduce impurities and structural defects, which can compromise GO’s suitability for dental applications [17,20,21]. Moreover, GO represents only one member of the broader family of GRM [16,22,23,24]. A key reason other GRM forms have seen limited adoption lies in their poor solubility and processability, often requiring additional chemical functionalization [23,25]. However, as the GRM family expands, top-down exfoliation routes have begun to shift the landscape by offering superior scalability and simpler processing. Another key advantage is the potential to use biomolecules to enable “greener” and safer synthesis methods [25,26].

A five-year literature analysis (Figure 1B), using search filters for GRM applications combined with biomolecules within and beyond the dental field, reveals that their integration into dental materials research remains limited, as indicated by the markedly lower publication volume compared to other domains. Biomolecules such as polyphenols, proteins, and polysaccharides have been employed to assist in GRM production, as they can exfoliate, stabilize, and functionalize graphene without significantly disrupting its sp^2^ network [25,27]. This results in fewer structural defects and aligns with goals to reduce environmental impact while tuning essential properties such as colloidal stability and biocompatibility [15,25,27]. The latter is particularly relevant in dentistry, where the clinical translation of graphene requires synthesis methods that meet both safety and regulatory standards [28].

To the best of our knowledge, the impact and growing relevance of biomolecule-assisted synthesis and GRM applications in dentistry have not yet been comprehensively reviewed. Therefore, this review aims to:

- Provide a framework of the GRM family and their synthesis routes;
- Review biomolecule-mediated graphene synthesis and its influence on physicochemical properties relevant to dental applications;
- Review current and emerging dental uses of GRM, identify key challenges, and present a roadmap to guide the selection of appropriate GRM forms depending on end use.

## 2. Framework of Graphene and Related Materials: A Focus on Liquid-Phase Exfoliation

**Figure 2:**
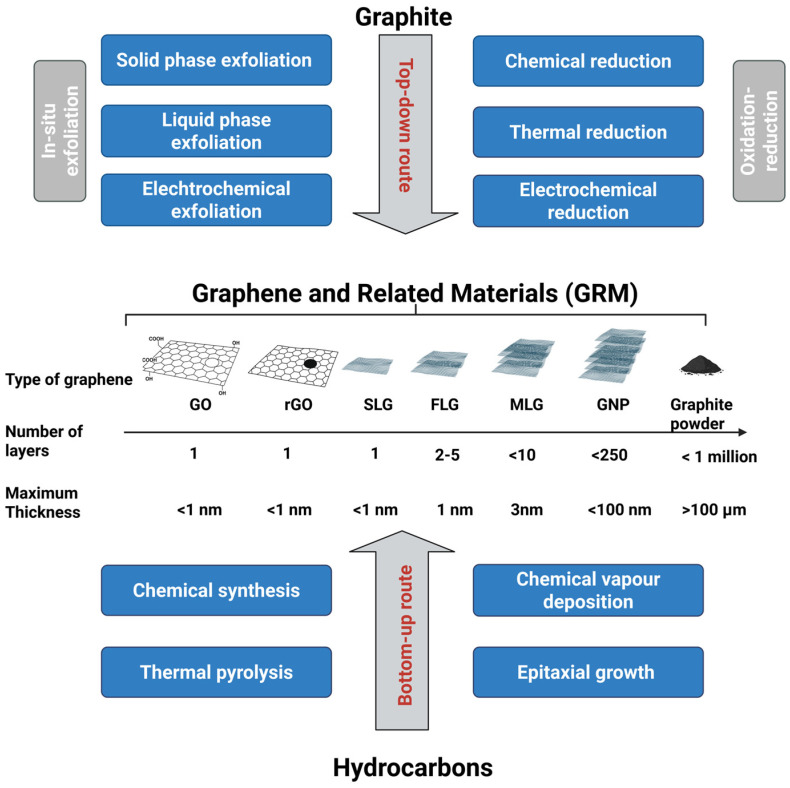
Schematic representation of the synthesis routes for graphene and related materials, including both top-down and bottom-up approaches. GRMs include: graphene oxide (GO), reduced graphene oxide (rGO), single-layer graphene (SLG), few-layer graphene (FLG), multilayer graphene (MLG), graphene nanoplatelets (GNP), and graphite.

GRM can be synthesized via two main approaches: bottom-up and top-down (Figure 2) [6,17]. Bottom-up methods like chemical vapor deposition (CVD) yield high-quality, large-area monolayers with excellent structural integrity [2]. However, their high cost, scalability issues, and substrate-transfer challenges limit practical applications [17,25].

In contrast, top-down methods, especially liquid-phase exfoliation (LPE), are more scalable and versatile, producing diverse GRM with tunable structural and surface properties [12,13].

Classifying exfoliated GRM is key to comparing results from different synthesis methods, as each process can yield materials with unique intrinsic properties [2,14,16]. The ISO framework defines GRM using key properties like layer number, defect level (I_D_/I_G_ where I_D_ and I_G_ represent the intensity of bands associated with defects and bands associated with ordered sp^2^ carbon atoms, respectively), oxygen content (oxygen to carbon ratio, O/C), crystallinity, surface area, sp^2^ lattice continuity, and flake size. Table 1 summarizes these features across main GRM types.

These differences affect how each material can be processed, functionalized, and applied [2]. High-quality forms like SLG offer excellent electronic and in-plane mechanical properties at the nanoscale but are difficult to produce at scale [2,6,16].

FLG retains many of these properties and can be obtained via liquid-phase exfoliation (LPE), offering a good balance between performance and scalability [6,15,23].

Oxidized forms like GO have higher dispersibility due to their high O/C ratio, but their synthesis often involves harsh solvents and results in increased defects and lower conductivity [2]. rGO restores some conductivity through chemical reduction, but this process introduces structural defects and impurities [2,6]. Mid-range materials like GNPs and MLG preserve the graphitic structure but are thicker and tend to aggregate [29,30]. Graphite, as a bulk material, lacks the 2D characteristics of exfoliated GRM [6,14].

**Figure 3:**
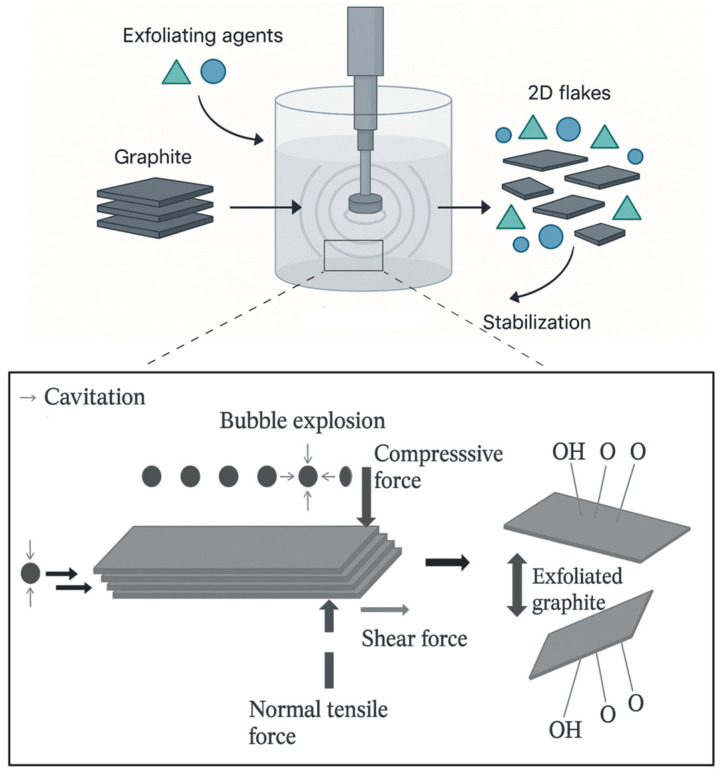
Schematic representation of liquid-phase exfoliation synthesis route.

In conclusion, FLG combines desirable SLG characteristics with the advantage of scalable production through LPE. LPE uses cavitation-induced forces to delaminate graphitic feedstocks into 2D flakes in appropriate solvents (Figure 3).

Since graphite is hydrophobic and prone to π–π stacking and van der Waals-driven restacking, exfoliating agents are essential to stabilize the dispersion [15,23,24,31]. These agents intercalate between layers, ensuring controlled delamination and colloidal stability [15,17]. While organic solvents are commonly used to exfoliate GRM, their high affinity for graphitic surfaces can interfere with later functionalization and leave behind toxic residues, which reduces their suitability for biomedical applications [15]. By contrast, aqueous exfoliation has attracted significant interest since water is a safe, low-cost, and environmentally friendly medium, positioning it as a promising approach for generating GRMs suitable for medical applications [15,23].

### 2.1. Overview of Biomolecule-Mediated Green Synthesis of Graphene Related Materials

Many non-natural molecules, such as pyrene derivatives and synthetic polymers, have been used to exfoliate GRM and improve suspension stability [15,32]. Exfoliation with aromatic molecules can give high yields, but the biosafety of the resulting flakes must be confirmed [15].

Biomolecules such as proteins, polyphenols and polysaccharides offer a safer alternative. Thanks to their low toxicity and natural origin, exfoliating GRM with bioactive biomolecules generally produces suspensions with better biocompatibility than those obtained using synthetic molecules [32,33,34].

These biomolecules intercalate between graphitic layers, improving exfoliation and preventing reaggregation through various adsorption mechanisms [32,33,34].Their distinct structures and interactions shape the final GRM physicochemical properties, meaning that processing conditions directly influence both final material quality and biological behavior [25]. Findings from GO cannot simply be applied to all GRM, as each form has unique structural profile that may trigger different biological responses [35].

Understanding how biomolecule-mediated synthesis shapes GRM physicochemical properties and biocompatibility is therefore essential.

#### 2.1.1. Polyphenols and Plant Extract-Assisted Synthesis

Polyphenols are a broad group of plant-derived molecules with strong hydrogen-bonding ability, metal-chelating activity, and redox properties. They interact with graphitic surfaces through π–π stacking on basal planes, hydrogen bonding at edge or oxygenated sites, and redox-driven partial reduction [36,37].

Tannin-rich extracts are among the most effective green exfoliants. Gallnut (*Quercus infectoria*) extracts, which contain high levels of hydrolyzable tannins (mainly gallotannins, 50–75%) together with gallic and ellagic acids, show strong antioxidant and antimicrobial activity against *Staphylococcus aureus*, *Escherichia coli*, and *Candida albicans* [38,39]. When incorporated into poly(vinyl alcohol) and chitosan matrices, these dispersions enhanced mechanical performance, raising Young’s modulus by up to 6.5-fold and tensile strength by up to 3.25-fold, with life-cycle assessment (LCA) indicating advantages over conventional solvent systems [32].

A second approach exploited the reductive properties of polyphenols to deoxygenate graphene oxide (GO) while preserving dispersibility. Using *eucalyptus* derived polyphenols, Saikumar et al. produced water-stable graphene by reflux-reducing GO, yielding 1–4 layer sheets with confirmed oxygen removal and long-term stability in various solvents [40]. Similarly, polyphenols from black tea (*theaflavins*/*thearubigins*) have enabled fast, scalable green exfoliation, producing graphene with low structural disorder (I_D_/I_G_ = 0.17) and improved compatibility with poly(methyl methacrylate) (PMMA) matrices [41].

Beyond tannins and tea catechins, phenolic-acid-rich extracts (chlorogenic-acid families: *caffeic*, *p-coumaric*, *ferulic*, *vanillic*, *p-anisic acids*) have also been explored as organic surfactants for GRM exfoliation and colloidal stabilization [18]. SEM, XRD and Raman analyses consistently showed few-layer structures with low defect levels. In one example, graphene/Parthenium nanocomposites displayed concentration-dependent antimicrobial activity, an effect absent in pristine graphene, indicating that the polyphenol layer modulates microbial interactions and enables tunable bioactivity [42].

Plant extracts containing mixed biomolecule systems can also act as effective stabilizers. Extracts from okra and baobab, which have hydrophilic–lipophilic balance values ≥ 8, support shear-assisted exfoliation and yield concentrated FLG dispersions (>1 g L^−1^). This approach is compatible with other 2D materials, including hexagonal boron nitride and carbon nanofibers, and the resulting dispersions have been processed into conductive films [43].

**Figure 4:**
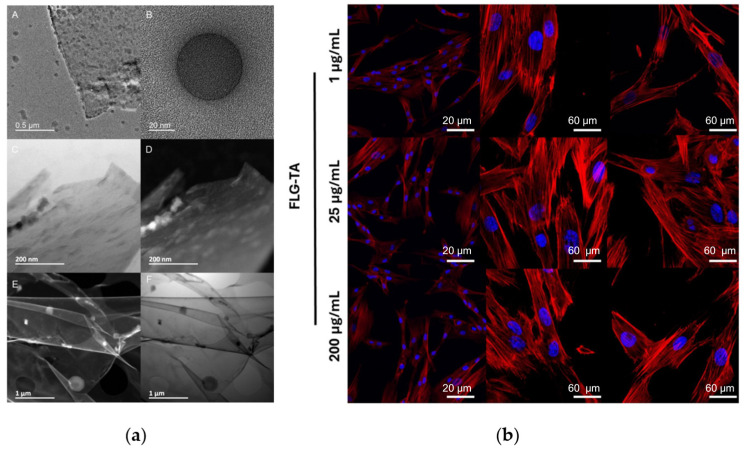
Tannic-acid–assisted liquid-phase exfoliation yields low-defect FLG and supports periodontal ligament cells biocompatibility: (**a**) Representative TEM micrographs of FLG layers obtained by TA-assisted liquid-phase exfoliation of graphite (**A**). Structure of spherical granules decorating the sheet surfaces (**B**). BF-STEM (**C**,**F**) and DF-STEM (**D**,**E**) images highlighting FLG sheets and particle distribution at the surface. BF: bright field; DF: dark field. (**b**) Representative merged confocal images of PDL cells exposed to different FLG–TA, showing phalloidin-stained F-actin (red) and Hoechst 33258-stained DNA (blue). Adapted with permission from Ref. [34]. Copyright 2025 Ben Ammar, T.; Kharouf, N.; Vautier, D.; Ba, H.; Sudheer, N.; Lavalle, P.; Ball, V.; licensed under CC BY 4.0.

Finally, individual polyphenols can form bioactive interfaces directly on graphene surfaces [34]. For example, tannic acid, a hydroxyl-rich polyphenol, enabled LPE-based FLG production, generating stable colloids (~2 µm lateral size, ~4 layers) with low defect density (I_D_/I_G_ = 0.16; Figure 4a). In addition to assisting exfoliation, tannic acid self-assembled into an oxygen-rich antioxidant coating that preserved radical-scavenging activity and showed good biocompatibility with periodontal ligament (PDL) cells (Figure 4b) [34].

#### 2.1.2. Polysaccharides

Polysaccharides are complex carbohydrates made of multiple monosaccharide units linked by glycosidic bonds. Among them, cellulose is the most abundant organic polymer in nature. Its structure, chains of D-glucose connected by β(1→4) linkages, gives it both hydrophilic (from hydroxyl groups) and hydrophobic (from C–H regions) properties. This amphiphilic nature allows it to act at interfaces, stabilizing water/oil emulsions [25,44,45].

This behavior has been used in LPE of GRM. Carrasco et al. showed that small amounts of cellulose nanocrystals can produce stable suspensions of mostly SLG. These suspensions are further stabilized by sulfate groups formed during acid hydrolysis [25,46].

Beyond cellulose, other hydrocolloid polysaccharides like maltodextrin and agar-agar have been mentioned in patents as eco-friendly dispersants for aqueous ultrasonication. They help form FLG-carbohydrate colloids [43]. By adjusting the ratio of solids/polymer to solvent, the texture of these formulations can be tuned from fluid inks (~5–30 g L^−1^) to gels or pastes (~30–80 g L^−1^). These mixtures can be redispersed in isopropanol, spread evenly on materials like textiles and foams, and form flexible, pressure-sensitive conductive coatings, making them promising for functional coatings [43].

Other polysaccharides like pullulan (neutral α-glucan), chitosan (cationic), and alginate (anionic) bind non-covalently to GRM surfaces and provide steric or electrostatic stability in water. Using bath sonication, they yield FLG with low defect levels (ID/IG ≈ 0.21–0.29) and varying oxygen content (C/O ≈ 7.2 for chitosan, 4.6 for pullulan, 4.1 for alginate). When applied to paper, chitosan–graphene achieved the lowest resistivity (~1.66 × 10^−3^ Ω·cm) and highest strain sensitivity (gauge factor ≈ 18.6). Alginate gave the highest graphene yield (~0.33 mg mL^−1^) and, along with chitosan, better colloidal stability than pullulan. These features make them well suited for flexible, paper-based strain sensors [47].

#### 2.1.3. Peptides and Proteins

Peptides and proteins act as biosurfactants surface-active ligands. These biomolecules support GRM water-based processing while adding functional groups and biocompatibility [48,49,50]. Peptides rich in aromatic amino acids, especially tryptophan, form strong π–π interactions with graphene surfaces, helping to peel layers apart and stabilize FLG dispersions [48,49,50]. Histidine-rich, lipid-modified peptides combine π–π stacking with hydrophobic interactions to produce stable aqueous suspensions and enable surface functionalization [48,49,50].

Among proteins, hydrophobins can form stable, non-covalent coatings that self-organize at hydrophobic surfaces, improving surface activity on produced GRM [51]. More broadly, the oxygen-containing groups on GO create hydrogen-bonding and electrostatic sites that interact well with proteins. These interactions can be tailored for specific uses such as molecular sensing, drug delivery, and tissue engineering [52,53].

Eco-friendly, high-concentration GRM dispersions can be achieved by sonicating graphite or expanded graphite in water, with or without shear mixing, using non-ionic proteins like bovine serum albumin (BSA), hemoglobin, or myoglobin. This method yielded stable graphene–protein colloids with few to multilayer flakes (up to 10 layers), typically at concentrations of 1–10 g L^−1^ and, under optimized conditions, up to 40–54 g L^−1^. Exfoliation yields range from 60–70%. The resulting flakes showed low Raman I_D_/I_G_ ratios, indicating minimal structural defects [43].

#### 2.1.4. Biocompatible Polymers

Poly(vinyl alcohol) (PVA) and poly(vinylpyrrolidone) (PVP) are synthetic, water-soluble polymers known for their high biocompatibility. PVA is a linear polyhydroxy polymer produced by hydrolyzing poly(vinyl acetate). It contains many hydroxyl groups, which enable strong hydrogen bonding and excellent film-forming properties [54].

PVP, in contrast, is a polyvinyl lactam derived from N-vinylpyrrolidone. Its pyrrolidone ring gives it amphiphilic character and outstanding solubility in both water and organic solvents [24]. Owing to these properties, both polymers are extensively applied in biomedical and pharmaceutical formulations, serving as stabilizers, excipients, and drug delivery carriers [55]. These same attributes, also make PVA and PVP highly suitable for assisting graphite LPE. A recent study demonstrated the synthesis of FLG using PVA and PVP as aqueous exfoliating agents. FLG/PVA was found to consist of flakes with 2–5 layers, a lateral size of approximately D90 ≈ 6 μm (SEM), and an I_D_/I_G_ ratio of 0.40 (Raman), indicating well-preserved structural features. In contrast, FLG/PVP exhibited similar thickness (2–5 layers) but larger lateral dimensions (D90 ≈ 10 μm) with an equivalent I_D_/I_G_ ratio of 0.40, highlighting the particularly favorable role of PVP in promoting the formation of larger graphene domains [56].

Findings from these studies confirm that the choice of biomolecule for in situ exfoliation is critical to the quality of GRM. Exfoliation yield, colloidal stability, and surface functionalization potential mainly depend on three intrinsic factors of biomolecules: molecular weight, charge distribution, and hydrophobic, hydrophilic, or amphiphilic character [15,43,57].

Low- to intermediate-molecular-weight biomolecules, such as polyphenols, are effective exfoliators. Their aromatic rings interact with graphene basal planes via π–π stacking, while hydroxyl groups bind to edge defects, supporting interlayer penetration, low viscosity, and high-shear exfoliation [22,26,34]. This dual mechanism results in high-yield, low-defect GRMs.

In contrast, high-molecular-weight polysaccharides and proteins act more as stabilizers. They bind electrostatically to graphene, preventing restacking [15,26,47,50]. However, their limited diffusivity reduces exfoliation efficiency compared to smaller molecules like polyphenols.

Charge also plays a key role:

- Anionic polysaccharides offer strong negative zeta potentials and long-term dispersion stability [58];
- Cationic agents like chitosan yield positively charged GRMs with stronger interactions but higher risks of non-specific adsorption [24,47];
- Neutral amphiphilic polymers (e.g., PVA and PVP) stabilize mainly through steric effects. They support high-solid-content dispersions but offer fewer reactive sites [15,55].

In summary, low- to moderate-molecular-weight biomolecules with aromatic or amphiphilic character are the most effective in LPE [43,57]. Polysaccharides, proteins and PVA/PVP are preferable when viscosity or biofunctionalization is needed [15,26,58]. A comparative summary is presented in Table 2.

Overall, biomolecules not only serve as sustainable exfoliants but also modulate key physicochemical properties that influence the biological performance of GRM.

The next section will explore how these properties relate to functional applications in the dental field.

## 3. Physicochemical Properties Dictates Function

### 3.1. Biocompatibility

Biocompatibility is paramount for dental applications where materials are in direct contact with dental tissues [28]. Studies show that GRM’s biological effects are strongly influenced by its physicochemical properties, such as concentration, lateral size, thickness, surface charge, hydrophobicity, and surface functionalization [35,60]. These properties can either increase or reduce toxicity [35,60,61]. For instance, GRM with many oxygen-containing groups is more water-dispersible, which helps reduce membrane disruption [62].

Functionalization also alters cellular uptake pathways: while pristine hydrophobic graphene tends to accumulate at the cell membrane, inducing reactive oxygen species (ROS) and apoptosis, functionalized hydrophilic graphene is internalized and induce less oxidative stress [61].

Likewise, graphene sheets and single-walled carbon nanotubes (SWCNTs) with similar chemical composition show different cytotoxicity effects due to variations in shape and aspect ratio, underlining the importance of morphology [35,62].

Biocompatibility of PVA/PVP-assisted FLG exfoliation showed that neither FLG/PVP nor FLG/PVA composites significantly affected the viability, apoptosis, or oxidative stress of THP-1 monocytes. At 25 μg/mL, FLG/PVA maintained THP-1 macrophage metabolic viability at around 90% compared to the untreated control. Even at 50 μg/mL, the formulation did not induce apoptosis [56].

A dose-dependent increase in IL-8 secretion was observed in monocytes exposed to either formulation. At 50 μg/mL, FLG also slightly increased IL-1β secretion in macrophages and elevated CD14 expression in monocytes [56].

The biocompatibility of GRM fillers also depends on whether the particles are free or embedded within a polymer matrix, which influences their interaction with surrounding tissues. In dental resins, for instance, the main source of toxicity is typically the leaching of unreacted monomers and fillers [13,63,64]. Post-processing steps, such as UV or thermal curing, generally increase the degree of conversion (DC), thereby reducing residual monomer release and improving biocompatibility [64].

In this context, incorporating GNP at appropriate concentrations and with proper dispersion can enhance the cross-linking density of the resin. This was demonstrated by increases in DC of approximately 14% in the as-printed state, 7% after UV post-curing, and 5% after thermal post-curing at 160 °C [64].

Mechanistically, GNP are believed to: (i) absorb incident light to promote photoinitiator radical generation, (ii) support energy or charge transfer to accelerate polymerization, and (iii) act as nucleation sites for cross-link formation [11]. Together, these effects lead to a denser polymer network, reduced monomer leaching, and improved biocompatibility. In tests across various GNP loadings (0, 0.025, 0.1, 0.5, and 1 wt%) in SLA/DLP 3D-printed resin, no cytotoxicity was observed in either L929 fibroblasts or gingival stromal cells (GSCs) after 24 or 72 h of incubation [63].

### 3.2. Bioactivity and Tissue Remineralization

Bioactivity in dental materials refers to their ability to stimulate beneficial biological responses within adjacent tissues, particularly through promoting mineral deposition [65,66,67]. Remineralization specifically pertains to the restoration of calcium and phosphate ions to demineralized dental substrates, a key treatment approach for managing tooth decay and mineral loss in teeth [66]. GRMs can directly enhance mineral deposition and osteogenic responses due to their high specific surface area, which promotes ion adsorption, and their tunable surface chemistry, which allows targeted functionalization, making them effective scaffolds for biomolecule immobilization [8,68,69,70].

Specifically, the carbon-to-oxygen (C/O) ratio and the spatial arrangement of oxygen-containing functional groups (such as carboxyl, hydroxyl, and epoxy groups) create calcium-binding sites that function as nucleation templates for the formation of calcium phosphate phases [71,72,73]. These interfaces could mimic the functional role of specialized non-collagenous proteins (NCPs) naturally present in mineralized dental matrices. Similarly to dentin phosphoprotein (DPP) and dentin sialoprotein (DSP), they create organized nucleation sites that control both the initial crystal formation and subsequent directional growth of crystals, processes essential for achieving remineralization [67,69,71,72,73,74].

A previous study showed that a dicalcium phosphate-reduced graphene oxide (DCP–rGO) composite significantly increased alkaline phosphatase activity and enhanced extracellular calcium deposition in pre-osteoblast cultures, indicating a synergistic effect of DCP and rGO in promoting osteogenic differentiation [75].

### 3.3. Antioxidant Properties

Oxidative stress is a key driver of pulpal inflammation and contributes to the cytotoxicity of various dental materials, especially restorative and endodontic cements. GRMs exhibit antioxidant properties that can help reduce oxidative damage and support inflammation resolution at the restoration–tissue interface [76,77].

**Figure 5:**
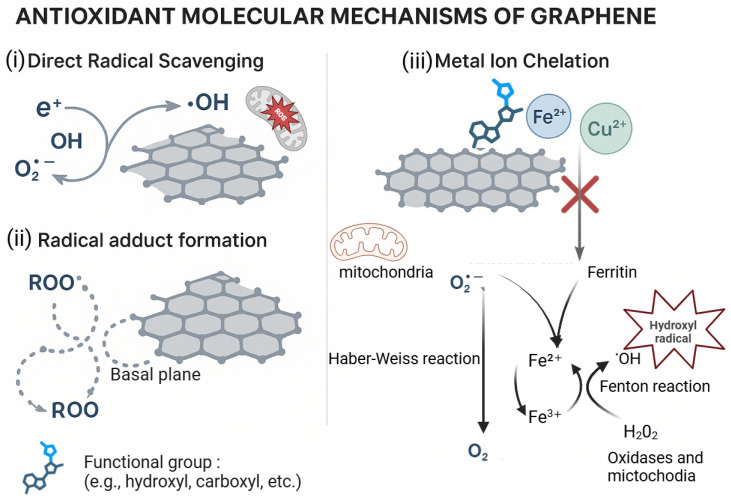
Antioxidant molecular mechanisms of graphene.

GRM’s antioxidant activity stems from its extended sp^2^-conjugated lattice, which gives it an inherent capacity to scavenge free radicals. Two main mechanisms are involved (Figure 5): (i) covalent bonding of reactive oxygen species (ROS) to defect sites or basal planes, forming stable radical adducts; (ii) electron or hydrogen donation to neutralize radicals such as •OH, O_2_•^−^, and peroxyl species.

These effects are enabled by high charge carrier mobility and a delocalized π-system [76,77,78]. Functionalized GRM (e.g., with hydroxyl, carboxyl, or polyphenol groups) can chelate Fe^2+^ and Cu^2+^, preventing the Fenton reaction, which otherwise generate highly reactive hydroxyl radicals from H_2_O_2_ [76,79].

In GRM–polyphenol composites, GRMs can act as supports for oxidized polyphenols and facilitate proton-coupled electron transfer (PCET), regenerating active antioxidant species and thereby extending ROS scavenging activity [79,80]. However, due to their non-diffusible nature and large surface area, these composites primarily function as stationary scavengers at interfaces. Their effectiveness depends on factors such as the C/O ratio, defect density, flake size, ligand density, and the balance between covalent stability and electronic coupling [41,80].

Notably, FLG–tannic acid (TA) exhibited significantly different antioxidant behavior in suspension compared to solid films: free TA in FLG dispersions quenched approximately 90% of radicals, while FLG-TA films on substrates scavenged only about 15% of DPPH radicals [34]. In a separate study, GO, at concentrations between 300 (ng/mL) and 60 (μg/mL), exhibited neuroprotective effects in SH-SY5Y human neuroblastoma cells by mitigating 6-hydroxydopamine (6-OHDA)-induced cytotoxicity and demonstrating ROS scavenging activity [81].

### 3.4. Antibacterial Properties

Dental caries, caused by the progressive demineralization of hard dental tissues due to cariogenic bacteria, is the leading cause of pulpal and periapical disease requiring endodontic treatment [82,83].

The goal of endodontic therapy is to prevent microbial contamination of the root canal system and eliminate existing pathogens to achieve successful clinical and radiographic outcomes [84]. However, even with thorough chemo-mechanical preparation, significant portions of the canal walls remain untouched, making complete microbial eradication practically impossible [85]. To overcome this limitation, various irrigant agitation techniques, such as pipetting, heating [86], and ultrasonic activation, have been developed to improve irrigant penetration [87].

Incorporating antibacterial agents into irrigants and endodontic materials is another strategy aimed at reducing residual microbes and preventing reinfection [88].

**Figure 6:**
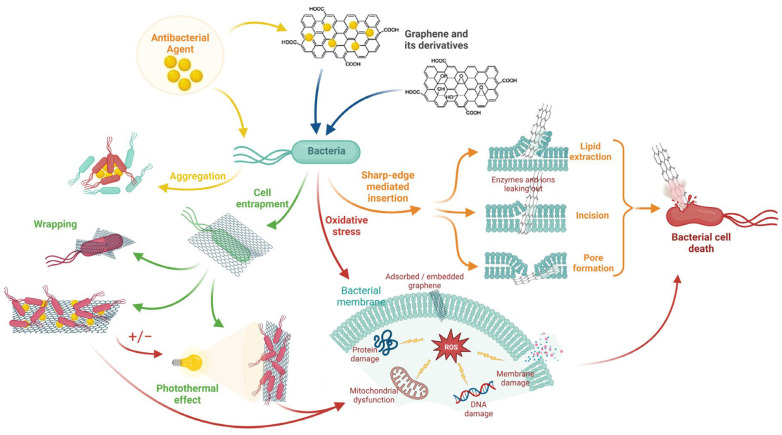
Antibacterial mechanisms of graphene (cell entrapment, oxidative stress, sharp-edge insertion). Reprinted with permission from [89]: Apostu, A.M.; Sufaru, I.-G.; Tanculescu, O.; Stoleriu, S.; Doloca, A.; Ciocan Pendefunda, A.A.; Solomon, S.M.; licensed under CC BY 4.0.

GRM have shown strong antibacterial properties through three main mechanisms: (i) the “nano-knife” effect, where sharp sheet edges disrupt bacterial membranes; (ii) oxidative stress induction in microbial cells; (iii) physical encapsulation of bacteria, blocking access to nutrients and inhibiting growth (Figure 6) [9,42,82,88,89,90].

The antibacterial activity of GRM has been evaluated in various models. GO nanosheets showed dose-dependent inhibition of oral pathogens such as *Streptococcus mutans*, *Fusobacterium nucleatum*, and *Porphyromonas gingivalis*. Complete inhibition of *P. gingivalis* and *F. nucleatum* occurred at 40 μg/mL, while higher concentrations (80 μg/mL) were required for *S. mutans*, consistent with membrane disruption and cytoplasmic leakage as key mechanisms [9]. Similarly, graphene-reinforced titanium (Ti-0.125G) showed significant antibacterial activity, especially against *P. gingivalis*. This effect may result from electron transfer from the bacterial biofilm to the graphene-titanium interface, disrupting microbial respiration and reducing viability [91].

In conclusion, antibacterial efficacy varies notably across studies, highlighting the influence of the physicochemical properties of GRM. Standardized characterization and further fundamental research, including comprehensive screening against clinically relevant oral microorganisms, are needed to clarify structure-activity relationships [9,90,92,93].

### 3.5. Mechanical Properties

**Figure 7:**
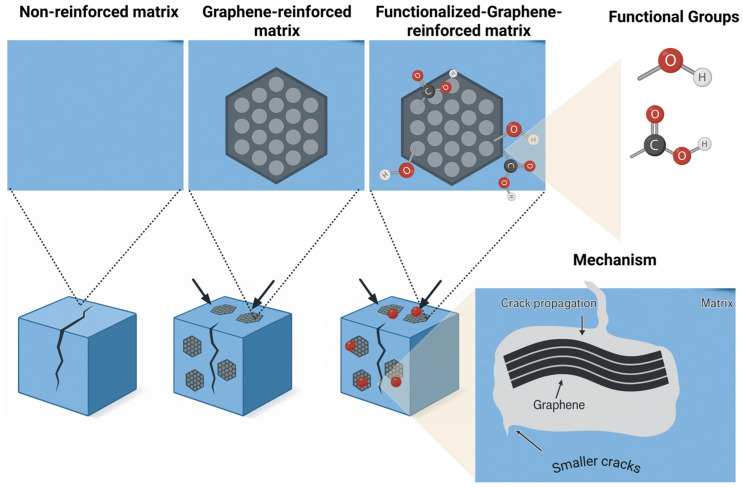
Mechanical reinforcement mechanism by graphene and related materials.

SLG demonstrates outstanding mechanical properties, including a Young’s modulus of approximately 1.1 TPa [94,95]. Although these nanoscale properties are difficult to fully translate at the macroscale due to agglomeration, low loadings of GRM can significantly enhance the mechanical performance of composites. Small additions to resin matrices have been shown to improve tensile strength, flexural strength, and fracture toughness by distributing stress and hindering crack propagation (Figure 7) [93,96].

To achieve these benefits, maintaining optimal filler loading is essential to avoid exceeding the percolation threshold. High dispersibility and uniform distribution are key [95]. GRM have also proven effective as reinforcing agents in dental polymers, ceramics, improving wear resistance and tolerance to masticatory forces through their intrinsic hardness and ability to restrict dislocation motion [94,95].

Functionalization could further enhance interfacial adhesion and dispersion [95]. Their inclusion also lowers the coefficient of thermal expansion (CTE), which is critical for the long-term stability of dental restorations under thermal stress [64].

Advanced methods like laser-assisted 3D printing of graphene-based structures highlight GRM’ growing role in next-generation dental materials [97].

## 4. Dental Applications

### 4.1. Endodontic Materials

Endodontic materials include sealers and obturation cements (epoxy, *zinc*-oxide–eugenol, and calcium based silicates/“bioceramics”), repair and retrograde cements for perforations and apical surgery, pulp-capping/plug materials, and auxiliaries such as irrigants and intracanal medicaments. These materials must perform in moist, chemically aggressive canals during cleaning, shaping, and obturation, as well as in root-end filling, perforation repair, apexification/apexogenesis [84,98].

Required properties for a durable sealing include dimensional stability, low solubility, radiopacity, bioactivity, antibacterial action, dentin adhesion, and workable handling/setting [82,98].

In available materials, shortcomings persist: microleakage (porosity/shrinkage), slow set and moisture-related washout, brittleness and low fracture toughness, limited sustained antimicrobial effect, discoloration (due to some radiopacifiers), challenging retreatability, and degradation after irrigant exposure [82,99].

Within this context, graphene-modified calcium-silicate systems are explored to enhance sealing, mechanical integrity, and antibacterial performance [100,101,102,103].

Incorporating reduced graphene oxide (rGO) into calcium silicate (CS) increased hardness by 40%, elastic modulus by 52%, and fracture toughness by 123%, with toughening via crack bridging, branching and deflection, collectively improving resistance to crack propagation [104].

Because calcium-silicate “bioceramic” sealers share hydration chemistry and microstructure with Portland cement, insights from graphene-modified cementitious composites are directly applicable to bioceramics [67,68]. Thus, graphene oxide (GO) serves as a nucleation scaffold that accelerates hydration, refines calcium–silicate–hydrate (C–S–H), and bridges microcracks, yielding denser matrices with higher stiffness/strength and reduced shrinkage and flaw propagation [68,69]. Mechanisms documented in white Portland cement composites translate to dental formulations as gains in compressive/microhardness, improved sealing against fluid percolation, and greater fatigue/wear resistance at root-end fillings, perforation repairs, and sealer–dentin interfaces under functional loading [105,106].

In bioactive cements, graphene nanosheets (GNS; 1, 3, 5, 7 wt%) tested in Biodentine (BIO) and Endocem Zr (ECZ) affected setting time, hardness, push-out strength, pH, cell proliferation, and mineralization; at 1–3 wt% GNS, both cements showed reduced setting time, increased hardness, and enhanced mineralization, with post-set composition assessed and bonding effectiveness highlighted as a point for further study [107].

### 4.2. Adhesives

**Figure 8:**
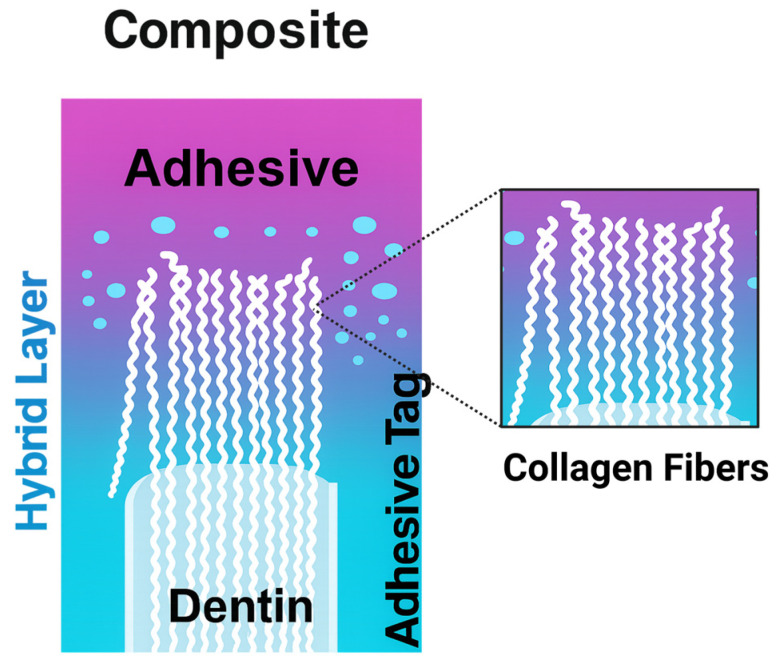
Hybrid layer formed at the adhesive-dentin interface.

The clinical success of dental restorations is strongly influenced by the quality of adhesion to dentin and the long-term stability of the hybrid layer formed during adhesive application (Figure 8) [108].

Although direct restorative materials offer excellent esthetics and high surface hardness, they remain constrained by polymerization shrinkage, interfacial degradation, and limited antimicrobial activity [8,108].

Indirect restorative systems achieve more reliable adhesion through chemical primers, most notably silanes for glass ceramics and 10-MDP for zirconia and hydroxyapatite, which form stable phosphate or siloxane bonds and thus provide stronger and more durable interfaces [109].

Despite these advances, conventional adhesive systems still face challenges in maintaining interface durability, predisposing restorations to secondary caries, debonding and thus reduced longevity [110].

To address these shortcomings, recent studies have investigated the incorporation of GRM fillers into dental adhesive systems. GO-modified primers increased the shear bond strength, surface roughness, and wettability of ZrO_2_ [52]. Similarly, Hydroxyapatite-GO composites demonstrated optimal performance at 2 wt% GO, yielding superior dentin bond strength and thermal aging stability [111]. In contrast, functionalized graphene systems, such as SiO_2_-Ag-Gr and HA-Ag in bis-GMA matrices, showed excellent biocompatibility (129% cell viability at 48 h vs. 53% for controls) but did not significantly enhance bond strength [112].

Complementing these experimental findings, computational work has also highlighted the intrinsic adhesive potential of GRMs. A molecular dynamics investigation evaluated how grain boundaries affect interfacial behavior in bi-crystalline graphene/polyethylene nanocomposites [13]. The results showed that the elevated energy at grain boundary regions enhances interactions at the nanocomposite interface, and that structural irregularities such as wrinkles and ripples further improve adhesion between the graphene nanofiller and the polymer matrix [13].

Despite these encouraging results, direct comparison between studies remains difficult due to variation in the type of GRM used, concentration ranges (0.05–2 wt%), resin composition, and dispersion protocols.

### 4.3. Restorative Materials

#### 4.3.1. Direct Restorations

Direct resin-based composites (RBCs) represent the most frequent choice in contemporary minimally invasive restorative dentistry, widely used for anterior and posterior restorations due to their excellent esthetics, adhesive capabilities, and minimally invasive application techniques [113].

They usually comprise a cross-linked methacrylate matrix, typically Bis-GMA, UDMA, and/or TEGDMA, filled with silane-treated inorganic particulates (e.g., silica, quartz, zirconia, or bioactive glass). Their macroscopic performance is governed by matrix chemistry, filler type/volume, and the quality of the filler/matrix interface [113].

Clinically, RBCs must tolerate cyclic occlusal loading and abrasive wear. Therefore, target RBC properties include high flexural/compressive strength, adequate fracture toughness, low polymerization shrinkage and stress, and resistance to hydrolytic aging [113].

While advances such as bulk-fill formulations and lower-shrinkage monomers mitigate gap formation during polymerization, current development increasingly pursues multifunctionality, materials that couple structural integrity with biological benefits (e.g., antibacterial activity or remineralization) via additives such as silver species, calcium phosphates, or quaternary ammonium compounds [113].

Within this paradigm, graphene based fillers have emerged as promising nanoscale reinforcers. Their high surface area and stiffness promote crack bridging and efficient load transfer, while oxygenated functional groups aid dispersion and chemical coupling to the resin network, limiting agglomeration [88,89].

Beyond this, GBMs display inherent antibacterial effects, offering dual reinforcement and biofilm control. Consistent with this, low GO loadings (≤0.2 wt%) have been reported to raise flexural properties and antibacterial efficacy without compromising handling. In a BisGMA/TEGDMA resin-cement system co-modified with graphene oxide (GO) and silver-doped hydroxyapatite (HA-Ag) for posterior restorations, increasing GO content led to higher flexural strength and compressive strength, while diametral tensile strength did not improve accordingly [114]. After 28 days, water sorption rose with GO loading, though surface morphology remained unchanged [114]. Antibacterial performance was strain-dependent, showing greater activity against *Escherichia coli* and *Staphylococcus aureus*, and was enhanced at higher GO and lower HA-Ag fractions [114].

#### 4.3.2. Indirect Restorations

Indirect restorations, including veneers, inlays/onlays, crowns, and fixed partial dentures, are fabricated extra-orally and luted with adhesive or conventional cements. Clinical success hinges on a demanding property profile: high flexural strength and fatigue resistance, wear compatibility with opposing dentition, color stability, and resistance to hydrothermal aging [115,116]. These materials must be biocompatible with gingival tissues, be resistant to wear and avoid wear of the antagonist tooth, and achieve precise marginal adaptation to limit bacterial colonization [115].

Over the past two decades, advances in ceramics have underpinned much of this progress. Glass-based systems (e.g., feldspathic porcelain, lithium disilicate) prioritize translucency and enamel-like esthetics, whereas polycrystalline ceramics (yttria-stabilized zirconia) deliver superior flexural strength and fracture toughness, making them preferred for high-stress posterior crowns and long-span prostheses [117].

In parallel, polymer-infiltrated ceramic networks (PICNs) or “hybrid ceramics”, combine an inorganic skeleton with an organic phase to enhance machinability, resilience, and shock absorption, suiting minimally invasive preparations and posterior inlays/onlays [118].

In addition, the digital manufacturing has reshaped workflows [119,120]. Subtractive CAD/CAM milling offers precise, reproducible restorations with standardized properties, reducing operator variability inherent to hand-layered techniques.

Additive manufacturing (AM) has emerged as a complementary paradigm: printable ceramics and hybrid ceramics are steadily improving, while stereolithography (SLA) and digital light processing (DLP) already enabling the direct printing of crowns, onlays, veneers [119,120]. Compared with milling, AM reduces material waste, accelerates prototyping, and supports intricate internal geometries. Nonetheless, most printable resins of methacrylate/urethane dimethacrylate (UDMA) families still trail milled composites or glass-ceramics in flexural strength, wear resistance, and aging stability; residual monomer and interlayer interfaces can further limit longevity [119,120].

Accordingly, current indications largely center on provisional or short-term restorations, although ongoing improvements in formulations and post-curing are narrowing the gap [119,120].

Within this evolving landscape, GBMs are a particularly promising lever. Graphene, can strengthen polymer matrices via improved interfacial bonding and potentially reduce polymerization shrinkage [89]. Furthermore, GBMs could impart antimicrobial activity that helps curb biofilm formation on provisional and definitive resin-based restorations.

Early studies indicate that low GO loadings can enhance mechanical strength, wear resistance, and thermal stability in printable resins while preserving printability and esthetics [63,64].

A second, widely used biomaterial is polymethyl methacrylate (PMMA), the long-standing standard for denture bases and interim prostheses due to its affordability, esthetics, straightforward processing, and repairability [115].

Despite its ubiquity, PMMA suffers from low fracture toughness, limited impact and wear resistance, and a tendency to promote bacterial adhesion, the surface porosity of conventional processing encourages adherence of dental pathogens, contributing to denture stomatitis [121].

Numerous reinforcement and antimicrobial strategies have been evaluated, e.g., glass fibers, zirconia, TiO_2_ nanoparticles, and biocidal additives [122]. Among these, graphene-based additives are especially promising: even at low loadings, graphene has been shown to raise flexural and impact strength through efficient stress transfer and crack deflection, enhance thermal stability, temper polymerization shrinkage, and reduce adhesion of oral bacteria.

**Figure 9:**
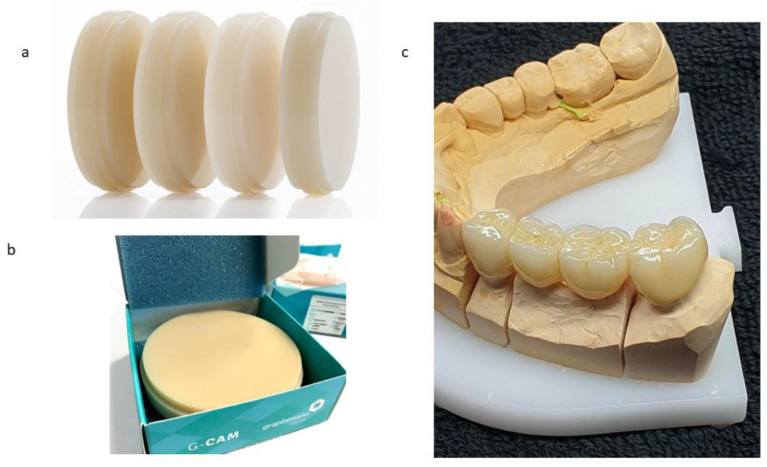
(**a**) Color range of PMMA–graphene offered by the manufacturer. (**b**) Samples studied as G-PMMA. (**c**) Dental restoration with G-PMMA with excellent esthetic results. Reprinted with permission from [121]: Punset, M.; Brizuela, A.; Pérez-Pevida, E.; Herrero-Climent, M.; Manero, J.M.; Gil, J.; licensed under CC BY 4.0.

At a very low filler loading (0.027 wt%), incorporating graphene nanosheets into polymethyl methacrylate (PMMA) yields the commercial composite G-CAM for dental prosthetic applications. Compared with neat PMMA, this graphene-modified biocomposite shows increased elastic modulus and compressive strength, alongside a marked reduction in specific wear rate, features that collectively enhance prosthesis durability and functional performance and excellent esthetic results (Figure 9) [121].

### 4.4. Cements/Luting Agents

**Figure 10:**
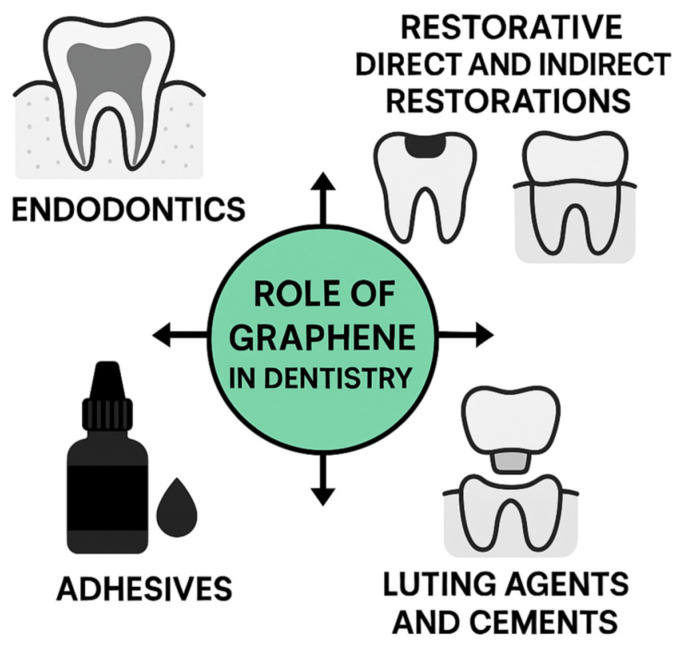
Role of Graphene-based materials in Dental Applications: Endodontics, Restorative Dentistry (Direct & Indirect), Luting Agents, and Adhesives.

Dental cements are indispensable for the long-term success of indirect restorations, providing mechanical retention, marginal sealing, and biological protection of the tooth–restoration interface (Figure 10) [123,124,125]. Conventional systems include zinc phosphate cements, valued for their historical reliability but limited by solubility and lack of adhesion; glass ionomer cements (GICs), which release fluoride and chemically bond to enamel/dentin yet show low strength; and resin-modified GICs (RMGICs), which combine improved mechanical properties with sustained ion release.

More recently, resin-based cements became the gold standard, especially for ceramic and CAD/CAM restorations, due to their superior bond strength, esthetics, and lower solubility [116]. Their performance, however, is strongly influenced by monomer composition (Bis-GMA, UDMA, TEGDMA), filler content, curing mode (light-, self-, or dual-cure), and adhesive strategy.

Clinical limitations remain, including polymerization shrinkage, hydrolytic degradation, marginal leakage, and vulnerability to recurrent caries [123,124,125].

In this context, the incorporation of GRM into these cements has attracted growing interest. Fluorinated graphene (FG) acted as a multifunctional modifier in glass ionomer cements (GICs), producing pronounced gains in hardness, compressive strength, wear resistance, and antibacterial activity without impairing fluoride release [116].

### 4.5. Protective Coatings

Metallic materials are widely used in dentistry, dental implants, orthodontic appliances, endodontic instruments, and prosthetic frameworks, but they remain susceptible to corrosion in the highly variable oral milieu (saliva, chlorides/fluorides, pH cycling, and biofilm-derived oxidants) [126,127,128,129,130,131].

Corrosion-driven ion release, notably of Ni, Ti, and Ag, can compromise mechanical integrity and raise biocompatibility concerns [132]. Although numerous surface treatments have been evaluated, particularly for nitinol (NiTi), conventional coatings show important drawbacks, including potential cytotoxicity, heterogeneous surface properties, inadequate adhesion and often exhibit surface irregularities, porosity, and delamination [126,127].

To address these challenges, GRM-based coatings have been proposed as an innovative solution. In fact, GRM exhibits great barrier properties, such as oxygen impermeability [133]. Furthermore, when enhanced with biomolecules like polyphenols, these coatings demonstrate remarkable synergistic effects, particularly in terms of antioxidant properties. The natural chelating capacity of polyphenols, especially tannins, has proven valuable in corrosion prevention since the 1990s [134].

Of particular interest are emerging self-healing systems that integrate GO with natural polyphenols and Zn^2+^. A recently reported nanoplatform based on *Tamarindus indica* extract and zinc ions (GON–Ti.E–Zn), incorporated into epoxy matrices, exhibited strong barrier behavior together with autonomous repair capability, providing an environmentally friendlier alternative to traditional chromate- and pyridine-based corrosion inhibitors [135]. Another study reported that a graphene nanocoating effectively protected titanium alloys from corrosion and ion dissolution over an extended period (240 days), maintaining excellent structural integrity and consistently high surface coverage (>98%) throughout all evaluation time points [132].

## 5. Conclusions

Across the reviewed studies, biomolecule-mediated exfoliation emerges as a sustainable, scalable, and clinically relevant method for producing GRM suited to medical contexts [15,32,34,43]. Liquid-phase exfoliation offers control over key parameters such as layer number, lateral size, defect density, oxygen content, and surface chemistry. However, the wide variety of GRM types makes material selection challenging, and comparisons between studies are often difficult due to different physicochemical characteristics [12,14,15].

While the recent technical specifications ISO/TS 9651: 2025 [136] and ISO 80004-13: 2024 [16] proposed revised nomenclature help standardize key structural elements and gives a framework for classification, a clear decision-making framework tailored to dental applications remains lacking.

To address this, we propose a practical roadmap to support the selection of specific GRM types based on their compatibility with the host matrix: In hydrophilic systems, such as MTA-type cements and sealers, materials like FLG, GO, or biomolecule-functionalized FLG are most suitable [101,102,103]. Their high oxygen-to-carbon (O/C) ratios and colloidal stability promote uniform dispersion and good dispersion in aqueous systems.

Additionally, these forms maintain chemical compatibility with hydration and setting reactions, making them preferable to more hydrophobic variants, which may impair curing behavior or phase stability. In contrast, hydrophobic systems, such as adhesives, resin composites used for direct restorations, CAD/CAM blocks, or 3D printing resins, could better benefit from GRMs like FLG, GNPs, or rGO. These materials offer superior dispersion in non-polar media and form stronger interfacial interactions within hydrophobic polymer matrices [12,63,125].

Compared to highly oxidized GRM like GO, which may disrupt polymer networks or increase moisture sensitivity, less functionalized GRMs (e.g., GNPs or rGO) enhance mechanical integrity and thermal stability [13,121]. Moreover, these GRM are commonly available in powder form, facilitating industrial integration using established dispersion techniques such as high-shear mixing or extrusion [13,121].

From a functional standpoint, GRM selection should be driven by the desired performance: mechanical reinforcement benefits from low-defect materials such as FLG or GNP. Their ordered lattice structures, indicated by low I_D_/I_G_ ratios (per ISO/TS 9651:2025) [136], enable effective stress transfer and improve fracture resistance. GRM content should remain below the percolation threshold to preserve mechanical integrity and avoid compromising biological performance [12,64].

For bioactivity and remineralization, GO or biomolecule-exfoliated GRMs, rich in functional groups, are more effective. These grafted groups mimic calcium-binding sites, enhancing cellular integration [65,103,110,137,138].

Antibacterial effects rely on oxidative stress generation and membrane disruption. GO or functionalized GRM with moderate to high defect densities (higher I_D_/I_G_ ratios) are especially effective. Functionalization with antimicrobial agents further boosts this activity, showing efficacy against oral pathogens such as *Streptococcus mutans* and *Porphyromonas gingivalis* [9,42,88,139].

For corrosion protection of metallic dental surfaces, defect-limited graphitic films, often produced via bottom-up methods, or thicker hydrophobic GNP provide effective barriers against ion leaching and degradation.

Biocompatibility, particularly for pulp- or dentin-contacting materials, is best ensured by biomolecule-assisted exfoliated GRMs, which avoid toxic residues from synthetic surfactants [15,56,99].

## 6. Future Prospectives

Ultimately, the development of biomolecule-GRM composites for dentistry should follow a mechanistic “synthesis → structure → function” framework. Key priorities include standardized synthesis protocols, harmonized physicochemical characterization, and durability testing under clinically relevant conditions. Long-term safety assessments, focusing on cytotoxicity and immunogenicity, are equally critical, alongside validated manufacturing and sterilization processes compatible with techniques like CAD/CAM and 3D printing. With these components in place, biomolecule-GRM composites have strong potential to transition from laboratory research to market-ready dental solutions.

## Figures and Tables

**Table 1:** Key strengths and limitations of intrinsic properties of graphene and related materials (GRM).

Form	Key Strengths	Limitations	References
**Single Layer Graphene (SLG)**	• High specific surface area • Minimal structural disorder (very low I_D_/I_G_) • Very high carrier mobility and low sheet resistance • High crystallinity and maximal in-plane mechanical properties	• High cost • extremely sensitive to contamination • difficult to scale up due to substrate transfer and low yields	[6,16]
**Few-Layer Graphene (FLG)**	• High lattice order with high sp^2^ character • lower intrinsic defects than GO or rGO • Scalable via liquid exfoliation • Offers potential for functionalization • Colloidal stability.	• Lower electronic properties as the number of layers increases, but still of interest • Reduced specific surface area compared to monolayer SLG.	[16,21,22]
**Multilayer Graphene (MLG)**	• High structural stability • Lower intrinsic defects than GO/rGO • Graphitic behavior with moderate conductivity • Scalable via liquid exfoliation • Offers potential for functionalization.	• Reduced “2D character”: electrical, optical and mechanical anisotropy • Lower specific surface area	[6,16]
**Graphene Nanoplatelets (GNP)**	• High lattice order with high sp^2^ character • High specific surface area relative to bulk graphite • Good crystallinity	• Increased flake thickness • Structural defects at platelet edges • Risk of aggregation	[16,29]
**Graphene Oxide (GO)**	• High oxygen content (increase in O/C) → high chemical reactivity • Hydrophilic surface • Colloidal stability.	• High structural disorder (high I_D_/I_G_) • Loss of sp^2^ network continuity → low electrical conductivity • Low crystallinity • high defect density in the basal plane	[16,21]
**Reduced Graphene Oxide (rGO)**	• Higher sp^2^ restoration vs. GO → improved conductivity • Intermediate oxygen content (decrease in O/C) • Higher crystallinity than GO.	• Residual oxygen groups → incomplete graphitization • Structural disorder (High defect level) • Properties depend strongly on reduction process → variability.	[16,21]
**Graphite**	• Highly ordered crystallinity in bulk form • Very low defect density within basal planes • Excellent thermal stability • Strong π–π stacking and interlayer cohesion	• No accessible 2D behavior (electronic/optical/mechanical) due to higher Thickness • Very low specific surface area • Poor dispersibility; strong tendency to restack • Limited surface reactivity (low oxygen content)	[6,16]

**Table 2:** Biomolecule-mediated exfoliation of graphene and related materials (GRM): summary of biomolecules and their nature, resulting GRM forms, methodologies employed, and key properties.

Biomolecule	Nature	Graphene Form	Method	Properties	References
Gallnut extracts	Polyphenols/ Hydrolysable tannins	f-FLG ^1^	LPE ^2^	Antioxidant Antibacterial	[59]
Eucalyptus Bark extracts	Polyphenols	rGO ^3^	Chemical Reduction	Long-term stability Good dispersibility	[40]
Black tea extracts (*theaflavins*/*thearubigins*)	Polyphenols	FLG	LPE	Additive (polymer reinforcement)	[41]
Tannic acid (TA)	Hydroxyl-rich polyphenol	FLG colloids (>1 g·L^−1^)	Shear-assisted exfoliation	Antioxidant good biocompatibility	[34]
Okra & baobab extracts	Plant extracts	FLG colloids (>1 g·L^−1^)	Shear-assisted exfoliation	Coating (conductive films/papers); Additive (ink base)	[43]
Cellulose CNC	Cationic amino polysaccharide	FLG	Bath sonication	Low resistivity; high strain sensitivity; stable colloids	[25,46]
Alginate, Chitosan	Polysaccharide/Anionic mannuronic/guluronic copolymer	FLG	Bath sonication	High yield; good stability	[47]
Aromatic-rich peptides (e.g., Trp ^4^-rich)/His ^5^-rich lipidated amphiphilic peptide	Peptides	FLG	LPE	Stable aqueous colloids; bio functional interfaces	[19,49]
BSA, hemoglobin, myoglobin (non-ionic proteins)	Proteins	Few- to multilayer graphene–protein nanocomposites (≤10 layers)	Aqueous ultrasonication ± shear	High-solid content; stable colloids	[25,43]
PVA ^6^/PVP ^7^	Biocompatible polymers	FLG	LPE	stable colloids	[24,54]

^1^ Few Layer Graphene; ^2^ Liquid-phase exfoliation; ^3^ reduced graphene oxide; ^4^ Tryptophane; ^5^ Histidine; ^6^ Poly(vinyl alcohol); ^7^ poly(vinylpyrrolidone).

## Data Availability

No new data were created or analyzed in this study. Data sharing is not applicable to this article.

## Abbreviations

The following abbreviations are used in this manuscript:

GRM	Graphene and related materials
ISO	International Organization for Standardization
TS	Technical Specification
SLG	Single-layer graphene
FLG	Few-layer graphene
MLG	Multi-layer graphene
GNPs	Graphene nanoplatelets
GO	Graphene oxide
rGO	Reduced graphene oxide
CVD	Chemical vapor deposition
LPE	Liquid-phase exfoliation
2D	Two-dimensional (materials)
LCA	Life-cycle assessment
I_D_/I_G_	Ratio of D-band/G-band intensities in Raman spectroscopy (with I_D_ = defect band intensity; I_G_ = graphitic band intensity)
PMMA	Poly(methyl methacrylate)
SEM	Scanning electron microscopy
XRD	X-ray diffraction
TA	Tannic acid
TEM	Transmission electron microscopy
BF-STEM	Bright-field scanning transmission electron microscopy
DF-STEM	Dark-field scanning transmission electron microscopy
BF	Bright field
DF	Dark field
PDL	Periodontal ligament cells
F-actin	Filamentous actin
DNA	Deoxyribonucleic acid
BSA	Bovine serum albumin
PVA	Poly(vinyl alcohol)
PVP	Poly(vinylpyrrolidone)
f-FLG	Functionalized few-layer graphene
CNC	Cellulose nanocrystals
Trp	Tryptophan
His	Histidine
ROS	Reactive oxygen species
SWCNTs	Single-walled carbon nanotubes
DC	Degree of conversion
UV	Ultraviolet
L929	Mouse fibroblast cell line L929
GSCs	Gingival stromal cells
THP-1	Human monocytic cell line THP-1
IL-8	Interleukin-8
SLA	Stereolithography (3D printing)
DLP	Digital light processing (3D printing)
NCPs	Non-collagenous proteins
DPP	Dentin phosphoprotein
DSP	Dentin sialoprotein
DCP	Dicalcium phosphate
PCET	Proton-coupled electron transfer
DPPH	2,2-Diphenyl-1-picrylhydrazyl (radical assay)
CTE	Coefficient of thermal expansion
CS	Calcium silicate
C–S–H	Calcium–silicate–hydrate
GNS	Graphene nanosheets
BIO	Biodentine
ECZ	Endocem Zr
10-MDP	10-Methacryloyloxydecyl dihydrogen phosphate
ZrO_2_	Zirconium dioxide
HA	Hydroxyapatite
SiO_2_-Ag-Gr	Silica–silver–graphene composite label (as used)
bis-GMA	Bisphenol-A glycidyl methacrylate
RT	Resin tags
AL	Adhesive layer
RC	Resin composite
RBCs	Resin-based composites
UDMA	Urethane dimethacrylate
TEGDMA	Triethylene glycol dimethacrylate
CAD/CAM	Computer-aided design/computer-aided manufacturing
PICNs	Polymer-infiltrated ceramic networks
AM	Additive manufacturing
G-CAM	Trade name for a graphene-PMMA composite
G-PMMA	Graphene-modified PMMA (as labeled in figure)
GICs	Glass ionomer cements
RMGICs	Resin-modified glass ionomer cements
FG	Fluorinated graphene
NiTi	Nickel–titanium (nitinol)

## Appendix A

### Appendix A.1

**Table A1:** Search strategy for the bibliometric analysis in PubMed and Web of Science, corresponding to the data presented in Figure 1A.

Search		PubMed	WoS
Per type of GRM	GO	(“graphene oxide”[tiab] AND (dentistry[tiab] OR dental*[tiab] OR “Dentistry”[Mesh] OR enamel[tiab] OR dentin[tiab] OR endodont*[tiab] OR periodont*[tiab] OR orthodont*[tiab] OR implant*[tiab] OR restorative[tiab])	TS = (“graphene oxide”) [AND (dentist* OR dental OR enamel OR dentin OR endodont* OR periodont* OR orthodont* OR implant* OR restorative)]
	rGO	(“reduced graphene oxide”[tiab] OR rGO[tiab]) AND (dentistry[tiab] OR dental*[tiab] OR “Dentistry”[Mesh] OR enamel[tiab] OR dentin[tiab] OR endodont*[tiab] OR periodont*[tiab] OR orthodont*[tiab] OR implant*[tiab] OR restorative[tiab])	TS = (“reduced graphene oxide” OR rGO) [AND (dentist* OR dental OR enamel OR dentin OR endodont* OR periodont* OR orthodont* OR implant* OR restorative)]
	SLG	(“single layer graphene”[tiab] OR “single-layer graphene”[tiab] OR SLG[tiab]) AND (dentistry[tiab] OR dental*[tiab] OR “Dentistry”[Mesh] OR enamel[tiab] OR dentin[tiab] OR endodont*[tiab] OR periodont*[tiab] OR orthodont*[tiab] OR implant*[tiab] OR restorative[tiab])	TS = (“single layer graphene” OR “single-layer graphene” OR SLG) [AND (dentist* OR dental OR enamel OR dentin OR endodont* OR periodont* OR orthodont* OR implant* OR restorative)]
	FLG	(“few layer graphene”[tiab] OR “few-layer graphene”[tiab] OR FLG[tiab]) AND (dentistry[tiab] OR dental*[tiab] OR “Dentistry”[Mesh] OR enamel[tiab] OR dentin[tiab] OR endodont*[tiab] OR periodont*[tiab] OR orthodont*[tiab] OR implant*[tiab] OR restorative[tiab])	TS = (“few layer graphene” OR “few-layer graphene” OR FLG) [AND (dentist* OR dental OR enamel OR dentin OR endodont* OR periodont* OR orthodont* OR implant* OR restorative)]
	MLG	(“Multi layer graphene”[tiab] OR “Multi-layer graphene”[tiab] OR MLG[tiab]) AND (dentistry[tiab] OR dental*[tiab] OR “Dentistry”[Mesh] OR enamel[tiab] OR dentin[tiab] OR endodont*[tiab] OR periodont*[tiab] OR orthodont*[tiab] OR implant*[tiab] OR restorative[tiab])	TS = (“Multi layer graphene” OR “Mult-layer graphene” OR MLG) [AND (dentist* OR dental OR enamel OR dentin OR endodont* OR periodont* OR orthodont* OR implant* OR restorative)]
	GNP	(“graphene nanoplatelet*”[tiab] OR GNP[tiab] OR GNPs[tiab]) AND (dentistry[tiab] OR dental*[tiab] OR “Dentistry”[Mesh] OR enamel[tiab] OR dentin[tiab] OR endodont*[tiab] OR periodont*[tiab] OR orthodont*[tiab] OR implant*[tiab] OR restorative[tiab])	TS = (“graphene nanoplatelet” OR “graphene nanoplatelets” OR GNP OR GNPs) [AND (dentist* OR dental OR enamel OR dentin OR endodont* OR periodont* OR orthodont* OR implant* OR restorative)]
	Graphene	graphene[tiab] NOT (“graphene oxide”[tiab] OR “reduced graphene oxide”[tiab] OR rGO[tiab] OR “graphene nanoplatelet*”[tiab] OR GNP[tiab] OR GNPs[tiab] OR “few layer graphene”[tiab] OR “few-layer graphene”[tiab] OR FLG[tiab]) AND (dentistry[tiab] OR dental*[tiab] OR “Dentistry”[Mesh] OR enamel[tiab] OR dentin[tiab] OR endodont*[tiab] OR periodont*[tiab] OR orthodont*[tiab] OR implant*[tiab] OR restorative[tiab])	TS = (graphen*) NOT TS = (“graphene oxide” OR “reduced graphene oxide” OR rGO OR “graphene nanoplatelet” OR “graphene nanoplatelets” OR GNP OR GNPs OR “few layer graphene” OR “few-layer graphene” OR FLG) [AND TS = (dentist* OR dental OR enamel OR dentin OR endodont* OR periodont* OR orthodont* OR implant* OR restorative)]
	Graphite	graphite[tiab] NOT (“graphene oxide”[tiab] OR “reduced graphene oxide”[tiab] OR rGO[tiab] OR “graphene nanoplatelet*”[tiab] OR GNP[tiab] OR GNPs[tiab] OR “few layer graphene”[tiab] OR “few-layer graphene”[tiab] OR FLG[tiab])AND (dentistry[tiab] OR dental*[tiab] OR “Dentistry”[Mesh] OR enamel[tiab] OR dentin[tiab] OR endodont*[tiab] OR periodont*[tiab] OR orthodont*[tiab] OR implant*[tiab] OR restorative[tiab])	TS = (graphit*)NOT TS = (“graphene oxide” OR “reduced graphene oxide” OR rGO OR “graphene nanoplatelet” OR “graphene nanoplatelets” OR GNP OR GNPs OR “few layer graphene” OR “few-layer graphene” OR FLG) [AND TS = (dentist* OR dental OR enamel OR dentin OR endodont* OR periodont* OR orthodont* OR implant* OR restorative)]

### Appendix A.2

**Table A2:** Search strategy for the bibliometric analysis in PubMed and Web of Science, corresponding to the data presented in Figure 1B.

Search		PubMed	WoS
Per Field	Biomolecule-mediated graphene integration in dental field	(graphene[tiab] OR “graphene oxide”[tiab] OR “reduced graphene oxide”[tiab] OR rGO[tiab] OR “few layer graphene”[tiab] OR “few-layer graphene”[tiab] OR FLG[tiab] OR “graphene nanoplatelet*”[tiab] OR GNP[tiab] OR GNPs[tiab] )AND( biomolecule*[tiab] OR protein*[tiab] OR peptide*[tiab] OR enzyme*[tiab] OR polysaccharide*[tiab] OR biopolymer*[tiab] OR “amino acid*”[tiab] OR DNA[tiab] OR RNA[tiab] OR “green synthes*”[tiab] OR biogenic[tiab] OR bioreduct*[tiab] OR “bio-reduct*”[tiab] OR “liquid phase exfoliation”[tiab] OR exfoliat*[tiab] )AND (dentistry[tiab] OR dental*[tiab] OR “Dentistry”[Mesh] OR enamel[tiab] OR dentin[tiab] OR endodont*[tiab] OR periodont*[tiab] OR orthodont*[tiab] OR implant*[tiab] OR restorative[tiab])	TS = ((graphen* OR “graphene oxide” OR “reduced graphene oxide” OR rGO OR “few layer graphene” OR “few-layer graphene” OR FLG OR “graphene nanoplatelet” OR “graphene nanoplatelets” OR GNP OR GNPs) AND (biomolecule* OR protein* OR peptide* OR enzyme* OR polysaccharide* OR biopolymer* OR “amino acid*” OR DNA OR RNA OR “green synthes*” OR biogenic OR bioreduct* OR “bio-reduct*” OR “liquid phase exfoliation” OR exfoliat*) AND (dentist* OR dental OR enamel OR dentin OR endodont* OR periodont* OR orthodont* OR implant* OR restorative))
	Biomolecule-mediated graphene integration beyond the dental field	(graphene[tiab] OR “graphene oxide”[tiab] OR “reduced graphene oxide”[tiab] OR rGO[tiab] OR “few layer graphene”[tiab] OR “few-layer graphene”[tiab] OR FLG[tiab] OR “graphene nanoplatelet*”[tiab] OR GNP[tiab] OR GNPs[tiab]) AND (biomolecule*[tiab] OR protein*[tiab] OR peptide*[tiab] OR enzyme*[tiab] OR polysaccharide*[tiab] OR biopolymer*[tiab] OR “amino acid*”[tiab] OR DNA[tiab] OR RNA[tiab] OR “green synthes*”[tiab] OR biogenic[tiab] OR bioreduct*[tiab] OR “bio-reduct*”[tiab] OR “liquid phase exfoliation”[tiab] OR exfoliat*[tiab]) NOT (dentistry[tiab] OR dental*[tiab] OR “Dentistry”[Mesh])	TS = ((graphen* OR “graphene oxide” OR “reduced graphene oxide” OR rGO OR “few layer graphene” OR “few-layer graphene” OR FLG OR “graphene nanoplatelet” OR “graphene nanoplatelets” OR GNP OR GNPs) AND (biomolecule* OR protein* OR peptide* OR enzyme* OR polysaccharide* OR biopolymer* OR “amino acid*” OR DNA OR RNAOR “green synthes*” OR biogenic OR bioreduct* OR “bio-reduct*” OR “liquid phase exfoliation” OR exfoliat*)) NOT TS = (dentist* OR dental)
